# Impacts of Lean Construction on Safety Systems: A System Dynamics Approach

**DOI:** 10.3390/ijerph16020221

**Published:** 2019-01-14

**Authors:** Xiuyu Wu, Hongping Yuan, Ge Wang, Shuquan Li, Guangdong Wu

**Affiliations:** 1 School of Management Science and Engineering, Tianjin University of Finance & Economics, Tianjin 300222, China; xywu2014@126.com (X.W.); lsq@tjufe.edu.cn (S.L.); 2 School of Management, Guangzhou University, Guangdong 510006, China; hpyuan2005@gmail.com; 3 College of Public Administration, Huazhong Agricultural University, Wuhan 430070, China; 4 Department of Construction Management, Jiangxi University of Finance and Economics, Nanchang 330013, China; wuguangdong@jxufe.edu.cn

**Keywords:** lean construction, safety system, system dynamics, construction projects

## Abstract

Lean construction has been viewed as an effective management approach for reducing the occurrence of no-value or destructive activities, such as wasting resources and safety-related accidents. However, few studies have systematically addressed how and to what extent lean construction practices influence construction safety. To bridge this gap, a conceptual model is developed and validated using a system dynamics approach. The construction system in this model comprises four sub-systems (i.e., environment system, equipment system, management system, and employee system). Data were collected from 448 projects in China. Simulations were conducted to determine the correlations between five types of lean tools and the four construction sub-systems. The results show that: (a) 5S management has significant positive impacts on the control of key locations and facilities at construction sites, and contributes to the mitigation of environmental impacts; (b) visual management can significantly improve safety compliance and safety management; (c) just-in-time management has significantly positive influences on the safety facilities layout and formulation of the safety plan; and (d) the Last Planner^®^ System and conference management are effective in improving safety training and the implementation of the safety plan. These findings provide new insights into the use of lean construction for improving construction safety through the implementation of a targeted lean approach.

## 1. Introduction

Construction projects, especially those building infrastructures, are booming in developing countries such as China, and have contributed greatly to social-economic development over the past few decades. Due to the complexity of the building environment during the construction stage, safety hazards are typically severe and pose enormous challenges to sustainable project delivery [1,2,3]. The safety levels of China’s construction industry are recognized to be deteriorating. According to statistics provided by the Ministry of Housing and Urban-Rural Development of China, there were 692 work-related accidents with 807 deaths on construction sites in 2017 [4]. In 2015, there were 442 accidents with 554 deaths [5]. In response, there has been a flurry of research on factors leading to safety hazards, as well as countermeasures to cope with safety issues, including the cause of accidents [6], safety inputs and safety performance theory [7,8], safety planning [9], safety culture, and safety climate and safe behaviors [10,11,12]. These studies provide significant theoretical guidelines for reducing the number of work-related accidents and hence improving safety performance. However, because of the “decentralized” and “mobile” nature of construction activities, as well as the “complexity” of the building environment, safety management measures that stem from the manufacturing industry are far from satisfactory in their application to construction projects [13,14].

The factors influencing construction safety performance have been widely analyzed, including work conditions, worker behaviors, and safety management systems [15]. With respect to work conditions, Winge and Albrechtsen [14] indicated that many accidents can be explained by the lack of physical barrier elements and that there is an urgent need to adopt a systematic barrier management approach. The subjects of safe behaviors involve owners, general contractors, subcontractors, and front-line workers on construction sites [16,17,18]. Safety programs that include safety commitments and responsibilities, safety supervisions, employee involvements, and safety evaluations are recognized as some of the most effective approaches for preventing construction work-related accidents [19]. Lean construction is a term that refers to systematic and value-added practices that can directly or indirectly overcome physical barriers and improve behavioral efficacy in construction safety management. More specifically, Bashir et al. [20] noted that implementation of the LPS (Last Planner^®^ System) and the 5S (seiri, seiton, seiso, seiketsu, and shitsuke) management method can significantly reduce accidents by tracking the roots of the problems. In a longitudinal study of 141 construction organizations, Thomassen et al. [21] found that the accident rate after implementing lean construction tools was 6.95% lower than in their absence. Furthermore, Forman [22] indicated that lean construction and the safety management system are two different types of social transformation “projects,” and that future research should consider how these two “project” types interact. Recently, the potential impacts of lean construction on safety performance have attracted increasing attention from scholars [23,24].

Koskela [25] first applied the lean thinking to the construction field and then put forward the concept of lean construction. Since the introduction of the Last Planner^®^ System [26,27], lean construction has rapidly developed and shown great effects on waste reduction, process control, and project value-adding [28,29,30]. However, the impact of lean construction on safety issues is as yet in its infancy and previous studies have been theoretical or involve just one type of lean tool. The influencing mechanism of lean construction on construction safety, therefore, lacks systematic and in-depth analysis. On this basis, this study focuses on five main types of lean tools—5s management, visual management, the Last Planner^®^ System, just-in-time management, and conference management—and investigates their impacts on improving construction safety. To address these concerns, in this study, we first developed a comprehensive model of the construction system (i.e., comprising environment, equipment, management, and employee sub-systems) through the lens of lean theories. Then, we targeted the optimization and improvement of the safety system from the perspectives of process control by providing a new perspective for improving safety management.

System dynamics, as proposed by Forrester [31], establishes a comprehensive model that comprises system structures, causal relationships, and feedback loops. Complex problems, such as causality, nonlinear relationships, multiple feedback, and time delay, can be addressed by applying system dynamics. Considering the advantages of system dynamics in simulating complex social systems, it has been widely applied to transportation systems [32], project management [33,34], and building design and operation [35]. As a kind of complex system, the construction safety system is characterized by multi-subjects, complex workflows, and a dynamic environment. As such, the system dynamics approach could be suitable to simulate the characteristics and operation process of construction safety systems.

The rest of this paper is organized as follows. First, a system dynamics model comprising five types of lean techniques is established. In Section 3, using the back propagation (BP) neural network and the mean impact value (MIV) algorithm, we verify the objectivity of the parameters within the system simulation process. The relationship between lean construction tools and the construction system is simulated in four scenarios in Section 4, wherein each scenario represents a construction sub-system. In Section 5, the study results are presented, including both the theoretical and practical implications.

## 2. Literature Review and Theoretical Model

### 2.1. Construction Safety System Model

The system accidents analysis has shifted from the cause-based analysis to system function analysis. System theoretic accident model and process (STAMP) is one of the most representative approaches. STAMP considers the influence of management commitment, culture, and feedback on safety performance [36]. A system consists of interrelated components (e.g., technical, physical, and human elements) and uses feedback loops of information and control to enforce safety constraints [37]. According to Grant et al. [38], complex systems need to identify the safety tenets through a systematical thinking approach to predict accidents. As for construction activities, the safety system involves people, materials, machinery, and activities related to safety issues [39].

**Figure 1:**
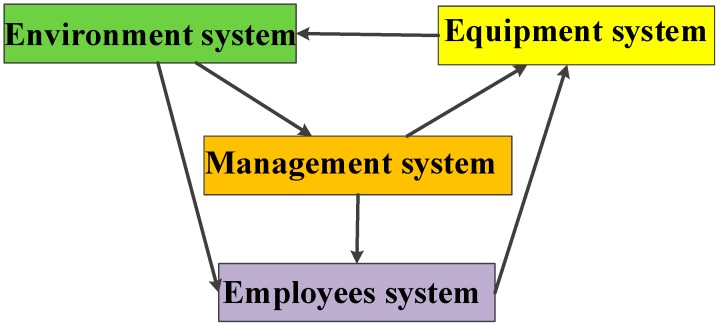
Structure and relationships of construction safety sub-systems.

To improve the operation of the construction safety system, scholars have conducted research from two perspectives. On the one hand, some researchers have focused on human issues, such as the behaviors of employees [40], safety management practices [41], and the relationship between the two [42]. Other researchers have addressed objects and environments, such as safety equipment [43] and safety environment [44]. As for construction projects, the safety system is in a continuous and dynamic process of change in which the elements intrinsically interact. To systematically analyze the dynamic effects of lean construction tools, in this paper, we divide the construction safety system into four sub-systems: the management system, employee system, equipment system, and environment system. Of these, the construction management (managers) system is the core sub-system [19], which has responsibility for the formulation and implementation of safety measures, safety inspections, and the correction of hazards. The employee (construction workers) sub-system includes employee safety participation (e.g., taking part in safety training) and safety compliance (e.g., compliance with safety regulations) [11]. The mechanical equipment and construction environment are peripheral sub-systems. The equipment system includes safety equipment and the equipment qualification rate, key parts, and control status of the equipment. The individual behaviors exhibited in the employee system directly affect the normal operation of the equipment system, while the management system indirectly controls the equipment system via the employee system. The environment system refers to the social and natural environment wherein the construction site is located and its influence on different project participants, including the owner’s satisfaction, disputes between managers and workers, and any public complaints about the construction environment [44]. In addition, the equipment system has a direct impact on the operation of the environment system and the environment system plays a significant role in the operation of the management and employee systems [43]. Figure 1 shows the structure and relationships of the four sub-systems.

### 2.2. Construction Safety System Operation Process and Its Feedback to Lean Construction Tools

Lean construction applies work structuring (alignment of product and process design and supply chains) to improve construction performance and workflow reliability. Moreover, lean construction provides the framework to ensure an orderly construction process and hence promotes the operating efficiency of the construction system [45].

**Figure 2:**
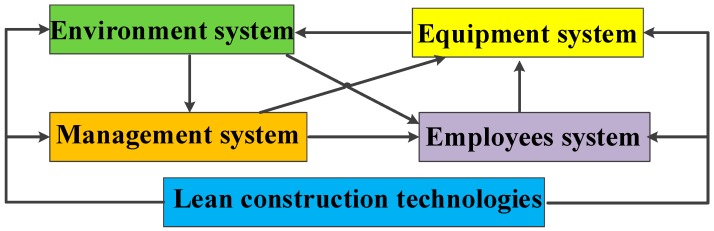
Construction system incorporating lean construction tools.

Figure 2 shows the operation and feedback of a construction system in a lean construction scenario. Lean construction includes a range of tools. This study mainly focuses on five specific lean construction tools, including 5S management, visual management, the Last Planner^®^ System, just-in-time management, and conference management. These five types of lean construction tools are closely related to safety practices and have gained wide attention in recent years, especially 5s management in occupational safety [46], visual management in construction safety [47], the Last Planner^®^ system with respect to safety involvement [48], just-in-time management regarding safety process [49], and safety meeting management procedures [50]. In addition, these lean construction tools have been widely used in China [51]. The following is a brief introduction to the five lean construction tools and the elements of the construction system.

#### 2.2.1. 5S Management

**Figure 3:**
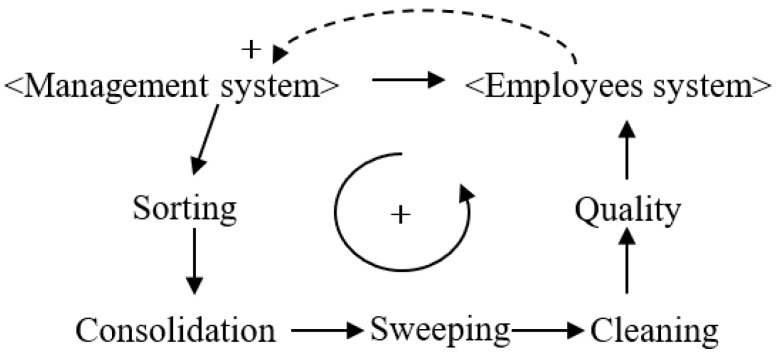
Construction system causality diagram based on 5S management.

5S management refers to the management of workers, materials, machines, and other on-site factors in the production process. Literally, 5S refers to the first letter of five Japanese words: sorting (seiri), consolidation (seiton), sweeping (seiso), cleaning (setketsu), and quality (shitsuke). Figure 3 shows a diagram of a construction system based on 5S management.

5S management was originally designed to reasonably optimize the layout of various on-site factors and thereby improve production efficiencies and reduce wastes. Thomassen et al. [21] noted that the implementation of 5S management would effectively reduce the number of work-related accidents caused by disordered management at the workplace, such as slipping, falling, and colliding. Specifically, “sorting” is performed to distinguish between necessary and unnecessary items found at the work site, and to then remove the unnecessary items and provide a more appropriate workspace. “Consolidation” is a direct result of sorting, whereby necessary items are arranged in order. “Sweeping” refers to cleaning up the workplace. “Cleaning” refers to maintaining the cleanliness of the workplace by its institutionalization and standardization. “Quality” refers to training workers to develop the cleaning habit. These five “s” steps are connected and complement each other. Sorting provides the basis for consolidation, and consolidation improves the sorting results. Sweeping embodies the effects of consolidation and sorting and all three steps form the basis for cleaning. The good “quality” of workers is achieved through the continued implementation and improvement in the above four steps. The establishment of worker quality eventually promotes improvement in the employee system, such that the construction system enters a virtuous cycle.

#### 2.2.2. Visual Management

**Figure 4:**
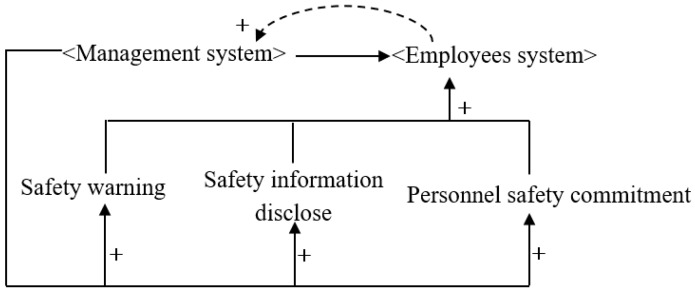
System causality diagram based on visualized management.

Visual management refers to managers using various tools or methods to achieve transparent and visualized management [52]. For example, the organization’s human resources policies, production norms, and processes are illustrated visually, so that workers can easily understand and follow its policies. After applying the visualized approach in safety management (e.g., safety warnings, safety information disclosure, and worker safety commitments), it is expected that those who lack safety consciousness and judgment will have nowhere to hide. Figure 4 shows a diagram of a construction system based on visual management.

#### 2.2.3. Last Planner^®^ System

**Figure 5:**
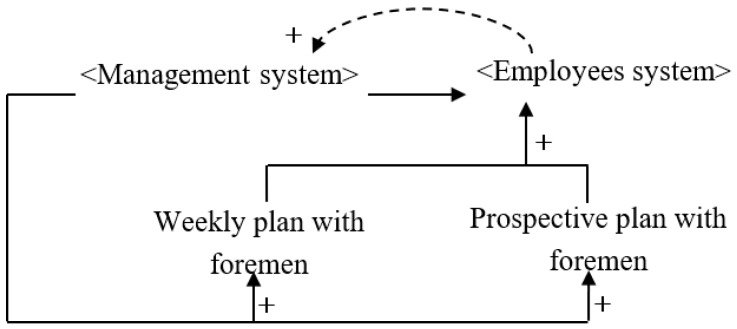
Construction system causality diagram for the Last Planner^®^ System.

The Last Planner^®^ System, also referred to as the Last Planner^®^, is a new pull-in construction planning and control tool that enables foremen at the frontline of the project (the last planner) to participate in project planning and monitoring to improve planning accuracy and reduce deviations [26]. However, research on its application to safety management remains scant. Tools for applying the LPS to safety management include the participation of foremen in the formulation of the weekly plan and the adjustment of monthly plans (prospective plans). On the one hand, implementation of the Last Planner^®^ arouses interest in safety participation by grassroots personnel, and on the other hand, it has the advantages of involving grassroots management personnel who are familiar with safety hazards at the construction site. Figure 5 shows a causal diagram of a construction system in the LPS.

#### 2.2.4. Just-In-Time Management

**Figure 6:**
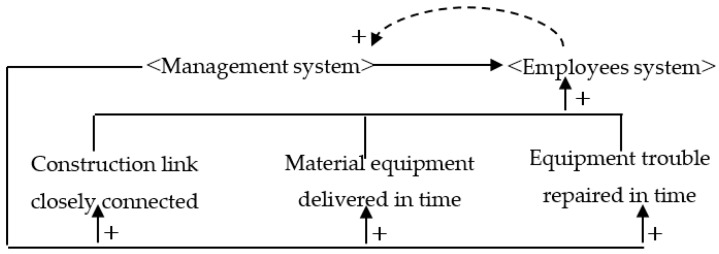
Construction system causality diagram for just-in-time management.

Just-in-time management (JIT) aims to reduce costs by optimizing inventory to enable the on-time supply of raw materials, semi-manufactured products, or finished products [53]. JIT management can shorten the waiting times of workers and the equipment. It has also been applied in the area of construction safety, with respect to the timely treatment of equipment failures and hidden safety hazards, the establishment of appropriate construction safety plans, and the elimination of periods of idleness. “Idle behavior” refers to the enforced idleness due to poor organization of work. Due to the defects of a construction management plan, workers have to spend more time waiting. As such, “idle” workers are likely to hang around the site and hence trigger work-related accidents. JIT management reduces safety hazards while also improving productivity. Figure 6 shows a causal diagram of a construction system in the JIT management scenario.

#### 2.2.5. Conference Management

**Figure 7:**
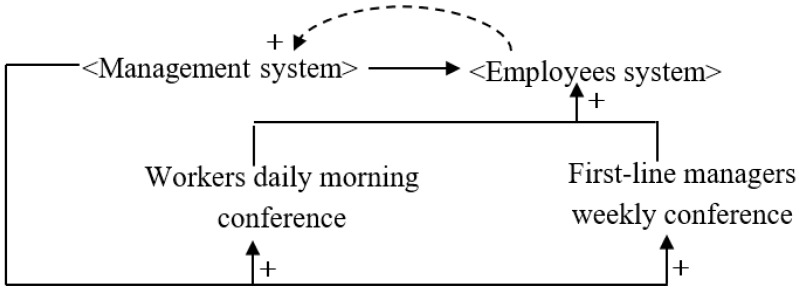
Construction system causality diagram for conference management.

Effective communication between workers and managers significantly reduces the number of work-related accidents, and conference management is reported to be the best way to communicate safety objectives [54]. Based on the lean construction concept, in this scheme, the foreman meets with construction workers each morning to discuss the work of the previous day and arrange the tasks for the day ahead. A weekly conference between the foreman and project manager is held to discuss problems that occurred during that week, which provides a good channel for solving problems at the construction site in a timely manner. It is notable that conference management is different from LPS. LPS emphasizes the participation of frontline workers in project planning and monitoring, while conference management focuses on establishing the way for communication and information sharing. Conference management not only involves the communications between project managers and foremen but also includes the daily huddles among project managers from different departments. Figure 7 shows a causal diagram of the construction system for the conference management scenario.

## 3. Methodology

### 3.1. Research Method

In this study, a hybrid research strategy was applied. First, the existing literature on safety management and lean construction was comprehensively reviewed to identify the operational processes of construction systems. This literature was retrieved from academic journals and industry reports and mainly focused on safety management and lean construction, such as safety planning [55], safety compliance [56], safety participation [17], and safety inspection [57]. This step provided the foundation for a system flow construct. From the systematic literature review, a questionnaire was developed, comprising two sections. The first section contained the respondent’s background information and the second included 24 items related to lean construction tools and construction safety systems. Details regarding the questionnaire survey process are provided in the data collection section. Next, to describe the safety level of construction systems, a quantitative model was developed, which includes parameter estimation and the establishment of equations. Finally, the system dynamics model was tested and a sensitivity analysis was conducted to explore the influence of lean construction tools on construction safety system. To verify the model, this study paid a return visit to some of the surveyed respondents and received their feedback on the analysis results of the system model.

In this study, the system model regarding the impact of lean construction tools on construction safety system considers the following three criteria:

Criterion 1: When project management measures are applied to the safety area, the basic requirement for the management system is that the implementation of the lean construction tool must be supported by safety management schemes and measures [25]. Thus, within the implementation process of a lean construction tool, a feedback node of the construction system must be established in the context of the management system.

Criterion 2: As a bottom-up change, the smooth implementation of lean construction lies in the positive safety participation of workers and their adherence to safety guidelines [43]. In addition to safety management support, lean construction also seeks workers’ feedback as a prerequisite of its implementation process. As a result, a feedback node for the lean construction tool and construction system is established in the employee system.

Criterion 3: Different lean construction tools have different emphases, and the cause and impact relationships differ in each sub-system. This study assumes that lean construction tools have a direct impact on each of the four sub-systems. The strength of these impacts is determined by the parameters used in the optimization calculation and simulation.

### 3.2. System Flow Construct

**Figure 8:**
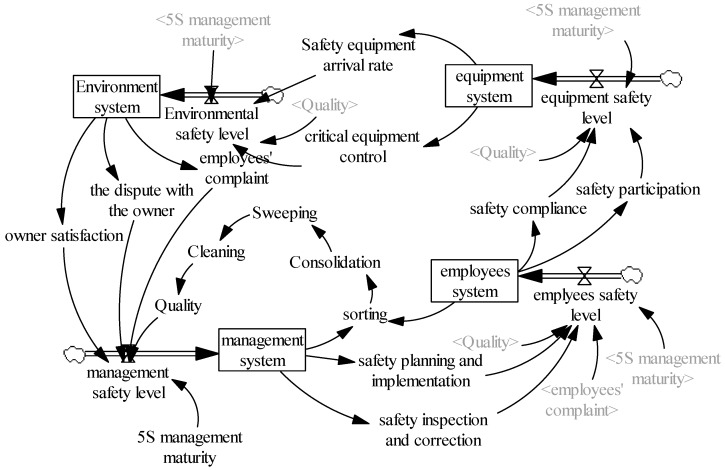
Construction system flow based on 5S management.

For each of the five types of lean construction tool, a construction safety system flow chart was established using the Vensim PLE system dynamics simulation software. In this study, we use the construction safety system flow chart for 5s management as an example to illustrate the analysis procedure (as shown in Figure 8). The horizontal variables include the four construction safety sub-systems—namely, the management, employee, equipment, and environment systems—and indicate the safety accumulation state of each in the system operation process. The rate variables refer to the safety level of each sub-system, which indicates the change rate of the horizontal variables. The auxiliary variables are the safety system and lean construction tools. For example, the safety plan formulation and implementation, along with the safety inspection and correction, refer to the output variables of the management system: sorting, consolidation, sweeping, cleaning, and quality, as represented in the 5S management tool. Regarding the other four types of lean construction tools, the principles of the construction safety system flowcharts are similar to that described for 5S management. In other words, the 5S management tool is replaced by the other lean construction tools to fix the structure of the safety system.

### 3.3. Data Collection and Validation

In this study, we investigated the implementation of lean construction and safety management measures in 448 projects, using a questionnaire survey to collect the data. A total of 24 items were included in the questionnaire. The questionnaire items include the implementation level of lean construction tools (15 items regarding the five types of lean construction tools) and the implementation level of the construction safety system (nine items regarding the four sub-systems). All the items were measured by five-point Likert scale, with points 1–5 indicating “strongly disagree” to “strongly agree,” respectively. At first, a total of 710 questionnaires were collected. 43 questionnaires were excluded based on the following criteria. (1) The number of missing items exceeds 10% of the total number of items; (2) The answers in the questionnaire are irregular (e.g., the same answer for all items). A total of 667 questionnaires were verified in terms of reliability and validity. A common method for reliability verification is to calculate Cronbach’s α coefficients. In this study, the Cronbach’s α coefficients of lean construction tools (fifteen items) and construction safety system (nine items) are 0.784 and 0.731 respectively, indicating satisfactory reliability and stability of the constructs [58]. Meanwhile, the validity verification was mainly implemented by content validation and construct validation. This study invited five project managers and three professors to discuss the content and expression of the questionnaire items through three rounds of group meetings. Based on the feedback, some flaws (e.g., vague expressions) in the initial questionnaire were further revised. The construct validation was tested in terms of Kaiser-Meyer-Olkin (KMO) coefficients. The KMO coefficients of lean construction tools and construction safety system are 0.824 and 0.794 respectively, exceeding the threshold of 0.6. Thus, the questionnaire was confirmed to be valid; and the data can be used in the subsequent analysis.

### 3.4. Parameter Estimation and Equation Establishment

The estimation of parameters, a key step in establishing a system dynamics model, provides the foundation for equation building and model simulation. In this study, the methods used for parameter estimation included trend extrapolation, regression analysis, table function, and historical experience methods. Most of these methods were used to determine parameter values based on existing functions or empirical decisions. To improve the objectivity and accuracy of the model simulation results, this study employed the back propagation (BP) network to calculate the mean impact value (MIV) as the correlation coefficient of each variable, thereby overcoming any subjective influence in the parameter determination.

**Figure 9:**
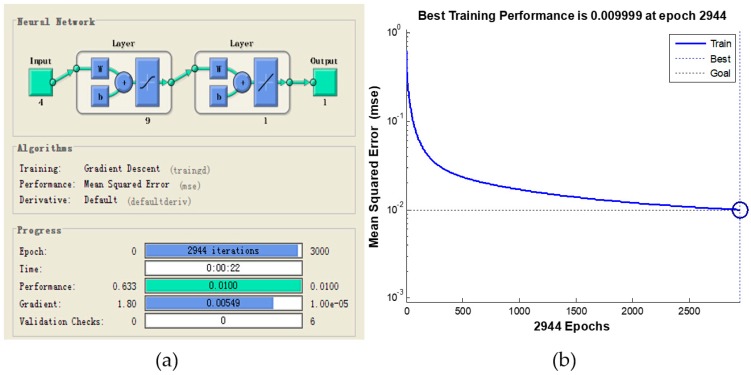
BP neural network topology structure (**a**) and simulation results (**b**).

Details of the parameter estimation process are as follows. First, a multi-input and single-output BP neural network was established according to the system flowchart. The input variables are the values of the influencing factors (i.e., independent variables) in the questionnaire, and the output variables are the values of the affected factors (i.e., dependent variables). Then, we used the MIV algorithm to calculate the effect of each influencing factor on the affected factors. Second, each of the independent variables in the training sample S was incremented or decremented by 10% from its original value, to obtain two samples S1 and S2. Third, using the network to simulate the S1 and S2 samples, we obtained the simulation results Q1 and Q2. The difference between Q1 and Q2 is the change value. The MIV value was obtained by calculating the average of the change value, whereby the greater the MIV value is, the greater the influence level of the independent variable on the dependent variable is. Using the above steps, the MIV value of each variable was considered to be the correlation coefficient between the influencing and affected factors, and the system dynamics equation was then established based on the correlation coefficient. The calculation of the MIV value of the safety management level for 5S management is used here as an example. According to the construction safety system flow chart for 5S management, as shown in Figure 8, we can see that the influencing factors on the safety management level include the owner’s satisfaction, disputes with the owners, and the number of complaints about the environment and quality. Thereafter, we used the four influencing factors as input variables in the BP neural network, with the safety management level being the output variable. According to the Kolmogarav theorem [59], the use of a single implicit layer can guarantee better accuracy of the fitting results for general function mappings. The number of implicit layer nodes is determined by the number of variables in the input and output layers (m = 2n + 1, where m is the number of implicit layer nodes, and n is the number of input nodes) [60,61]. Therefore, the number of implicit layers is one, and the number of implicit layer nodes is nine. Figure 9a shows the neural network topology structure. The ‘tansig’ and ‘purelin’ functions were selected in the transfer process of the implicit and output layer nodes, respectively. The maximum number of model iterations was set to 3000, with a target accuracy of 0.01 and a learning rate of 0.05. Figure 9b shows the model simulation result. When the iteration number was 2944, a target accuracy of 0.01 was achieved, and the MIV values of the four output variables were 0.33, 0.25, 0.26, and 0.16, respectively after normalization. Therefore, the system dynamics equation was established as follows: “management safety level = 0.33 × owner’s satisfaction + 0.25 × the dispute with owners + 0.26 × employees and public complaints about the environment + 0.16 × quality”. System dynamics equations for other variables were similarly established.

### 3.5. Model Testing and Validation

Prior to simulation and analysis, the validity of the system dynamics model must be determined. According to Sterman [62], the major testing criteria are as follows: (1) the variables in system causal loop flow depicting the lean construction and construction safety system must be aligned with the research statement; (2) the equations in the system causal loop flow must conform to the relationships between lean construction and the construction safety system; and (3) the system dynamics model must be consistent at all dimensions, and, ultimately, the model should pass the extreme condition test.

The first test criterion is that the model contains all the important variables that relate to the research purpose. This was determined by confirming the contents of the system causal loop flow. As shown in Figure 8, all the relevant variables are included in the flow and clearly represent the effects of lean construction on the construction safety system. The second test criterion is that the model is logical and reasonable, with reference to the system causal loop flow. In this study, the system flow is in line with the construction practice. The third test criterion is that the measurement units of all variables in the model are consistent at all dimensions, which was determined using a units check” action in the Vensim 5.7a software. Because all variables in the model were measured using a five-point Likert scale, the measurement units of the variables are consistent. In sum, the model passed the validity test and can be used for further simulation and analysis.

## 4. Simulation Analysis and Discussion

The distribution of the validated questionnaires covers 18 provincial areas, of which four provinces are located in north China (e.g., Beijing, Tianjin, Hebei, and Inner Mongolia) and accounted for 57.6% of the data. Four provinces are located in east China (e.g., Shandong, Shanghai, Zhejiang, and Jiangsu) and accounted for 33.2% of the data. The rest of the provincial areas accounted for 9.2% of the data. The project types in this study include civil buildings, industrial buildings, municipal facilities, and other projects, with respective percentages of 57.7, 13.6, 18.4, and 10.3%. The respondents included individuals in all job designations in construction organizations. The percentage of first-line managers and technical professionals was 83.2%, middle managers accounted for 14.1%, and senior managers accounted for 2.7%. The demographic characteristics of the projects and respondents are diverse and thus comprehensively reflect the condition of lean construction tools and safety management.

Since the duration of construction projects typically extends beyond one year, we performed a simulation analysis of the research model on a yearly basis for an overall period of five years. In addition, to analyze the influence mechanism of lean construction tools on the safety system, two maturity levels are adopted as control variables. Then, the influence level of different lean construction tools on the safety system at different maturity levels were compared and analyzed. A technical maturity level of 0.1 was recorded as “A” and that of 0.7 as “B.” The simulation analysis results are as follows.

### 4.1. Effects of Lean Construction Tools on Construction System

**Figure 10:**
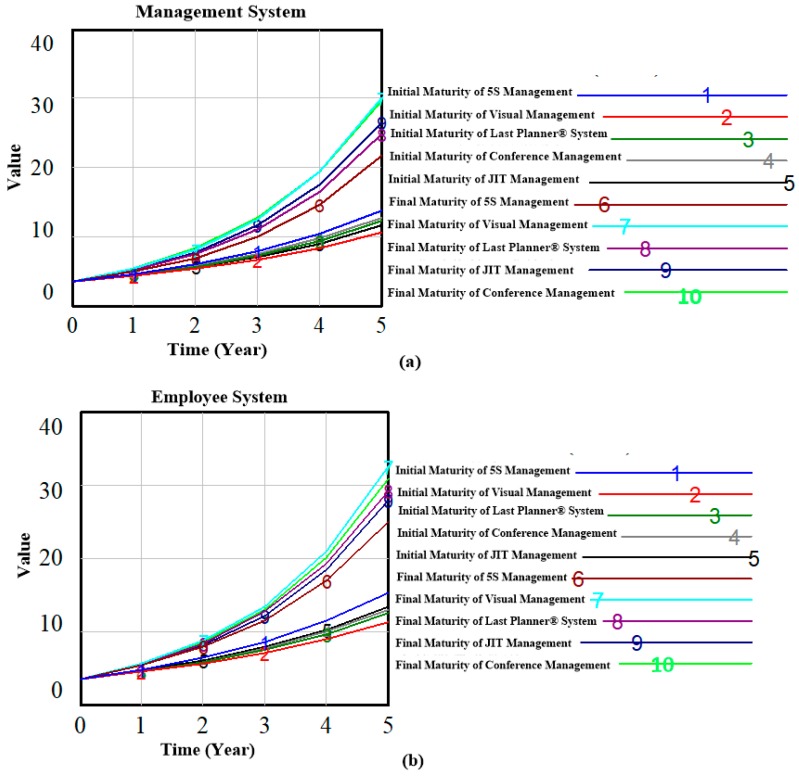

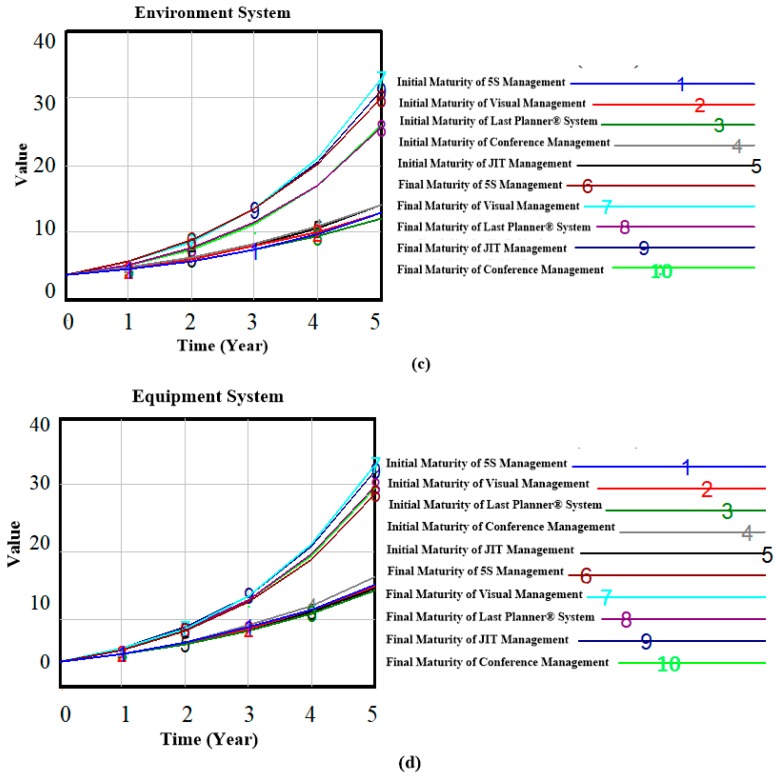
Construction system safety level under different lean construction technical levels: (**a**) Management System, (**b**) Employee System, (**c**) Environment System, (**d**) Equipment System.

In Figure 10a, the change trend of the safety level of the management system is shown by the changing maturity level of the lean construction tools. In the figure, we can see that when the maturity levels increase from 0.1 to 0.7, the safety level of the management system is significantly improved (lines “1” to “5” indicate the initial maturities of 5S management, visual management, the LPS, conference management, and JIT management, respectively, and then become lines “6” to “10”). Of these lines, those labeled “6,” “7,” “8,” and “10” improved more than the others, which indicates that the application of JIT management, visual management, conference management, and the LPS can significantly improve the safety level of management systems. Similarly, Figure 10b shows the change trend of the safety level of the employee system when the maturity level of the five types of lean construction tool is changed. In the figure, we can see that improvement in the maturity level of the lean construction tools can greatly improve the safety level of the employee system. The most significant effects were made by JIT management, conference management, and the LPS. Of the five types of lean construction tool, visual management, JIT management, and 5S management exhibited the most prominent impacts on the equipment system (as shown in Figure 10c). Figure 10d shows the influence mechanism of lean construction tools on the environment system, from which we can see that when the maturity levels of lean construction are changed, the environment system is significantly improved. No significant difference is observed between the degrees of improvement of the five types of lean construction tool. In other words, all five types are consistent in their effect on the safety level of the environment system.

### 4.2. Discussion and Implications

The sensitivity analysis results show that the five types of lean construction tool exert various influences on the construction system. This is due to the fact that different lean construction tools have different emphases with respect to construction activities. The influencing mechanism of the lean construction tools on the construction system is described in the following.

5S management primarily influences the equipment and environment systems. It deals with the emergence of unsafe conditions or equipment protection issues. With sweeping, sorting, and consolidation as its core steps, this tool aims to keep materials and equipment in excellent condition and to avoid any accidents caused by a disorganized work environment [21]. 5S management also improves the workplace and living environment by cultivating good working habits [63]. After contacting the surveyed project managers and workers, the effect of 5S management can be summed into the following two aspects. On one hand, the complaints (from workers or nearby communities) about the on-site working environment have been reduced; and on the other hand, the level of dissatisfaction of the owners has also been decreased.

Visual management mainly influences the management, equipment, and environment systems. It targets safety warnings regarding dangerous usage of equipment to enhance employee safety awareness and provides a platform for the dissemination and communication of safety management policies [64]. Visual management enhances the visualization of the on-site safety situation, while also improving the management and equipment systems, and avoiding safety disputes due to failure to fulfill safety responsibilities. The application of building information modeling to safety management makes visual management more influential with respect to construction systems [65]. After consulting the surveyed project managers and workers, it can be found that visual management urges them to keep an eye on changes in construction safety systems, and hence improve their intuitive understanding and memory.

The LPS primarily influences the management, employee, and environment systems. Mitropoulos et al. [66] noted that proactive planning is an effective way to cope with task uncertainty. The LPS provides a driving force for the formulation and implementation of safety management planning [24]; and it also enables frontline employees to participate in the formulation of work planning, which enhances their sense of responsibility and motivation, and therefore enhances the safety training and promotes their compliance with safety regulations and participation in safety activities. Finally, the role of the LPS is mainly reflected in the communication of the safety environment policy and participation in safety activities. The safety environment system is essential to the participation and support of frontline employees. Thus, it becomes an effective way to change the environment system from “push” to “pull.” By contacting the surveyed respondents, it can be found that LPS improves workers’ engagement as well as the operating efficiency of construction planning.

Conference management mainly influences the management, employee, and environment systems. Conference management is essentially a communication platform for resolving the problems that arise in daily construction activities. It provides a mechanism for managers and workers to regularly communicate about safety issues. In particular, the morning huddle enables workers to better understand and prepare for their work and to avoid safety hazards to a large extent. After contacting the surveyed respondents, it can be confirmed that the conference management is one of the most effective ways to communicate and share safety information.

JIT management significantly impacts all four sub-systems. First, it emphasizes the fact that rigorous construction inspection and the timely correction of safety hazards can promote the formulation and implementation of safety management planning [27]. Secondly, it improves the effectiveness of the safety schedule and avoids safety hazards caused by the worker idleness [67]. Thirdly, it emphasizes the timely elimination of hidden dangers in equipment, with the aim of preventing work-related accidents caused by the unsafe operation. Finally, it aims to prevent materials from being left and equipment from sitting idle at the construction site. Ideally, there are no idle workers, which also contributes to 5S management objectives (e.g., cleanliness) at the construction site. By contacting the surveyed respondents, it can be found that JIT management not only help save time and cost but improve the site environment and avoid the safety hazards due to the materials exposure on site.

In summary, lean construction tools have the most direct impact on improving workers’ safe behaviors, including their safety compliance and safety participation behaviors, and different lean construction tools show different effects on the construction system. In addition, it is notable that the implementation level of lean construction tools depends, to a large extent, on the support of managers.

## 5. Conclusions

Considering the increasing number of construction work-related accidents in combination with poor levels of safety management in rapidly developing countries, in this study, a construction safety system was developed based on five typical lean construction tools. Through the lens of system theory, logical relationships between the five types of lean construction tool and the safety system were identified, and a questionnaire was designed to collect data regarding the implementation level of safety systems and lean construction tools. Using the system dynamics approach, we analyzed the influencing mechanisms of lean construction tools on safety systems. The results indicate that the maturity level of lean construction tools plays a significant role in improving the safety level of a construction system. Interestingly, the five types of lean construction tool have different impacts on the four sub-systems: 5s management focuses on improving the equipment system (e.g., the control of key parts) and plays a crucial role in improving the environment system; JIT management and visual management have a significant effect on perfecting the management and equipment systems; the LPS and conference management enhance management and employee systems. Furthermore, these five types of lean construction tool all significantly influence the environment system. In sum, the implementation of lean construction tools has a significantly positive effect on improving overall safety systems. This study further analyzed the influencing mechanism of lean construction tools, as well as developed the research of construction safety from systematic thinking. The results of this study revealed that it is necessary to adopt different types of lean construction tools to cope with different safety issues, and also shed light on improving safety management levels and developing the safety management capabilities.

The limitations of this study are as follows. First, the surveyed participants all come from China. This sampling technique limits the generalizability of research findings to other geographic contexts. Whether the conclusions are applicable to different contexts needs to be further verified. Second, this study aims to reveal the influencing mechanism of lean construction tools. Noteworthy, in the course of the study, we found that the implementation barriers of lean construction tools attract increasing attention from the project managers. How to effectively promote the adoption of lean construction tools could be a potential direction for future research.
